# Supplementary material for: Exploring nine simultaneously occurring transients on April 12th 1950
Source: Sci Rep. 2021 Jun 17;11:12794. doi: 10.1038/s41598-021-92162-7 (PMC8211679; doi:10.1038/s41598-021-92162-7)
Supplement: Supplementary file 1 — Supplementary material 1 (pdf 12227 KB) [file 41598_2021_92162_MOESM1_ESM.pdf]

APPENDIX

A. APPENDIX

A.1. *Observations and data reduction:*

We obtained imaging observations with the SDSS r- and g- filters using the OSIRIS instrument (1; 2) at the 10.4m Gran Telescopio Canarias (GTC). These observations were carried out in the night from the 25th to the 26th of May 2020. 12 exposures of 150 sec each in the r-band and 9 exposures of 200 sec each in the g-band were obtained. The detector of OSIRIS consists of two 2048×4096 Marconi CCD44-82 CCDs with a $9.4''$ gap between them. By default, binning 2×2 is used, which corresponds to a pixel scale of $0.254''$. We reduced the OSIRIS imaging data with self-written IDL codes. The raw images were bias-subtracted and then flat-fielded with sky flats. The reduced images of each CCD were then combined separately: first we measured the position of one star in all images of each CCD to determine the shifts between the individual exposures. In the next step we aligned the images with integer pixel shifts (by simply rounding the measured shifts) and mean-combined them, also using sigma-clipping (with a cut of 10 σ) to get rid of cosmic rays. The images taken with the OSIRIS instrument reach depths of ~ 26 magnitudes.

A.2. *Astrometric corrections*

The accuracy of the World Coordinate System (wcs) kewords in the FITS headers is not enough for our purposes. To improve the astrometry we used the online tool at http://nova.astrometry.net/. We started by putting in the size, the coordinates and limits of the field as a first guess. The procedure by default also includes the computation of a SIP polynomial distortion correction with order 2. Due to some distortions of the original OSIRIS images, the astrometric solution delivered by the Astrometry.net tool is insufficient in some areas of the OSIRIS FoV.

In the next step, we refined the astrometric solution further using the TERAPIX software[2] (3), in particular with the SCAMP and SWarp tools. A global astrometric solution for each individual CCD scan projected onto a common system was computed with comparison to the SDSS DR13 catalog for absolute calibration.

A.3. *SExtractor analysis of astrometry-corrected images*

In order to estimate the chance projection probabilities, we needed to first estimate the number of objects in one image. The GTC counterparts to the transients were visually identified (all being independently visible in both the g and r images) using DS9. Now, the question was how many objects of the same luminosity or brighter were present in the images. In order to estimate the number, the source lists were obtained using SExtractor (4). We adopted the default parameters given in the configuration file except for *DETECT THRES* which was modified to keep sources with 3σ or brighter magnitudes above the background. Using the channel 1, g image, SExtractor identified 2157 sources.

A.4. *Identification of counterparts and photometry of counterparts*

For the counterparts we have previously identified visually with the help of DS9, we perform photometry to obtain the magnitudes. The photometric calibration of Osiris images refers to SDSS photometry and SDSS standard fields. In order to detect the sources in the reduced images, we used SExtractor in single-image mode. We detected sources in g and r bands with signal-to-noise (S/N) ~ 3 in at least 10 adjacent pixels, which implies a 1.5σ detection threshold in the filtered maps. We used MAGAUTO mode and elliptical aperture photometry. Detected sources were then correlated with corresponding sources from the SDSS catalog (using the matching radius of $1''$) and, thus, the zeropoint for each band was obtained. With S/N ~ 3, we detect sources in one band up to $g \sim 25$ and $r \sim 26$. In order to obtain photometry for weaker objects, we created a few extra catalogs with lower signal-to-noise. We note, however, that this threshold concerns the identification of a counterpart in one band, while the counterparts we list in Table 1 are detectable in both the r and g bands *independently*, which of course increases the total S/N (and certainty) of each detection.

beatriz.villarroel@su.se

[2] http://terapix.iap.fr/soft/

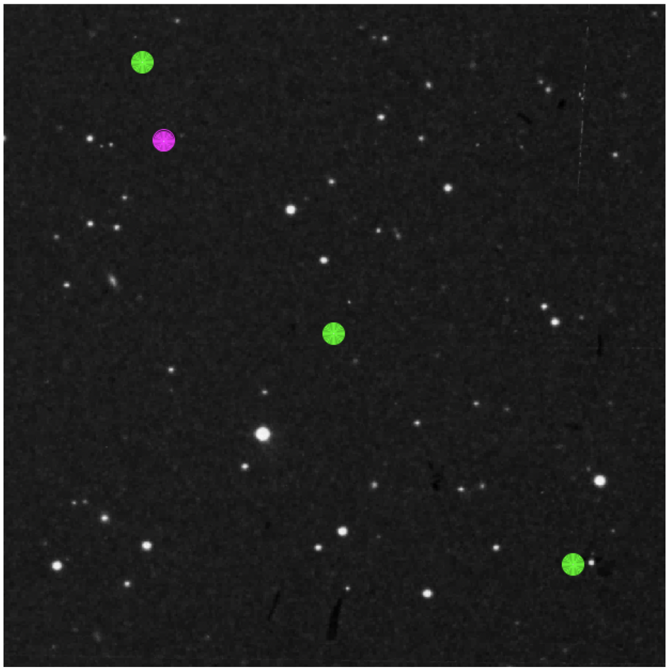

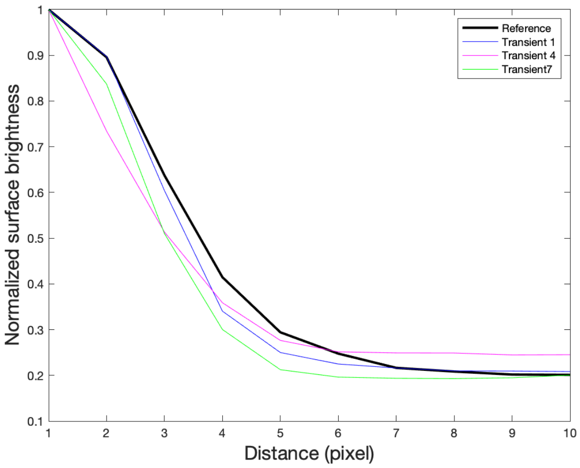

Figure 1. Examples of radial profiles. In the left figure, we show the example stars. The one in purple is a real reference star, those in green are 3 of the 9 transients. In the right figure, we show the radial profiles of the example star and the selected transients.

A.5. *PSFs of transients*

Judging from Figure 1 in the Letter, it is clear that the 8 out of 9 transients are very star-like point sources. In Figure 1, we show the radial light profile of a few of the transients by measuring the surface brightness with the "annulus" function in DS9. We also show the radial profile of a reference star. Since photographic plates have a non-linear response, we chose a reference star that has more or less similar brightness to the transients in Figure 1. We can see transients have similar shapes on the light profiles as normal stars.

We note, that in Table 1 of the Letter, we calculate the FWHMs with an automated procedure in (5), which may give different results than the manual DS9 approach.

We also show the comparison to other bands in Figure 2.

A.6. *Automatized detection of more simultaneous transients*

By chance, we identify a bright transient outside our region of the sky at (ra,dec) = 212.5714,27.0909 (see Fig 3). We estimate roughly that it has an apparent magnitude around $r \sim 12$ mag, which means at least an 11 magnitude drop in luminosity.

This means that the XE325 plate, that is about $6 * 6$ square degrees in size, may have more transients detected than only those in the crowded region that are the focus of our Letter. Naturally, since the plate is rather large, there must be a good number of observed transients. In order to search for more transients on the XE325 plate, we must obtain several reference plates. We contacted the STScI archive and asked to obtain XE325, the plates taken right before (XO325, the blue plate at the same coordinate) and the plate observed right after (XE384). The XO325 and XE384 plates were used for the tests in Section A.7.4. We also asked to retrieve the red plate that includes our field of interest, but was taken 6 nights later (XE324). The plates involved are listed in Table 1.

In order to identify more transients on XE325, we ran the photometry procedure (5) on XE325 and XE324, the two photographic red plates that show the same sky region 6 nights apart. The overlapping region between XE324 and XE325 is located inside the region $212 < \mathrm{Ra} < 213$ and $26 < \mathrm{Dec} < 32$. Slight cuts on the edges of the plate were made, which exclude the bright transient at (Ra, Dec) = 212.6284,26.9782. The photometry code produces a list with equatorial coordinates (J2000, epoch = 1950), full width half maxima (FWHM), instrumental magnitudes and r magnitudes in the Gaia system for each object on the photographic plate.

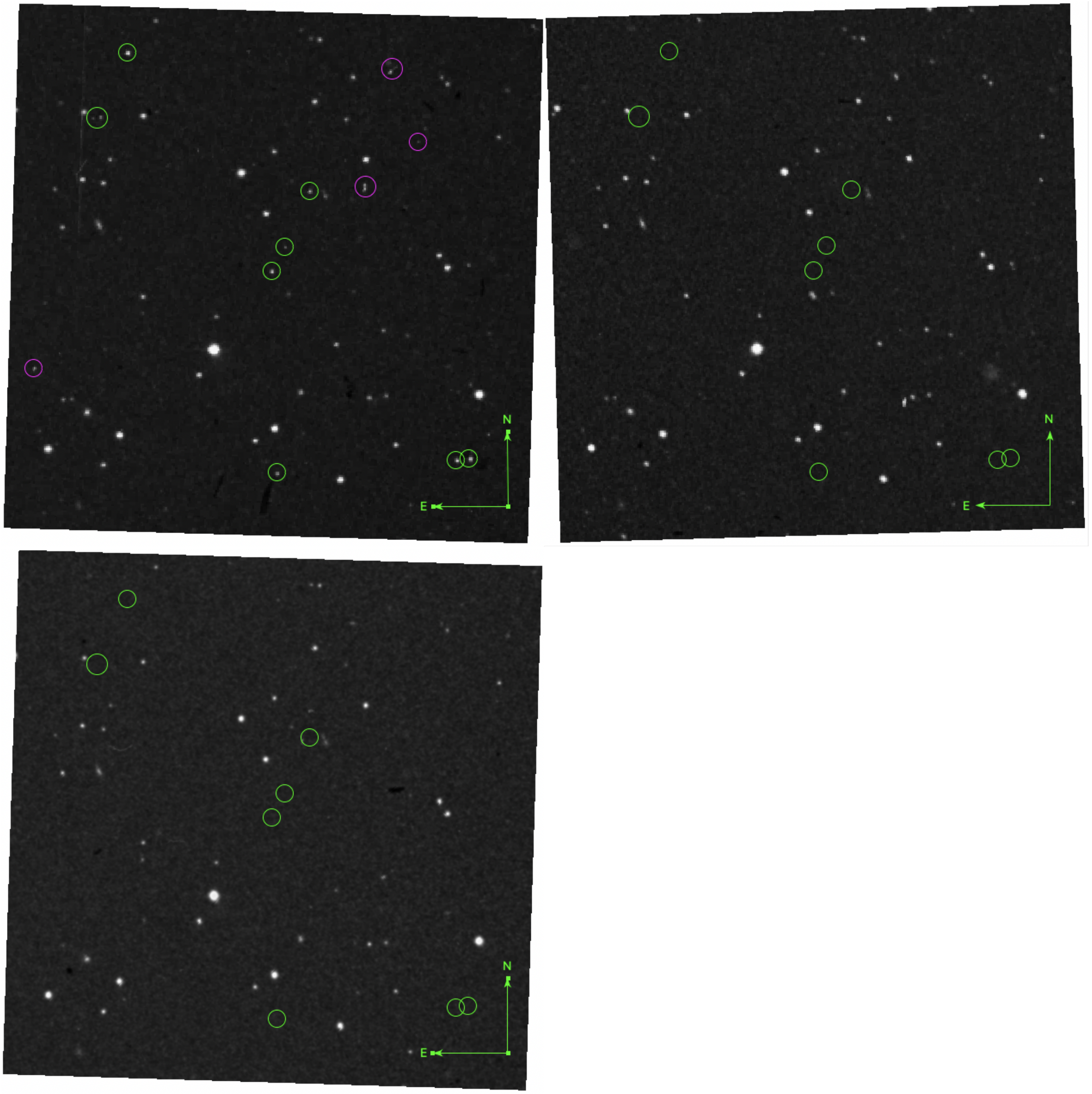

Figure 2. The interesting 10 x 10 arcmin^2 field shown in other bands. In the POSS-1 (left, upper) image we see a number of objects that cannot be subsequently found, observed on April 12th 1950. The right, upper image shows another POSS-1 red image taken six days later on April 18th 1950. Below, we show the blue POSS-1 taken half an hour earlier on 12th of April 1950. Purple circles in the 12th of April red (upper left) image are artifacts from the scanning process. Small artifacts can be found at random places in all images, distinguishable by their non-smooth, non-circular shapes. About nine sources are present in the POSS-I E image from the 12th of April 1950, but not in any other POSS images.

By cross-correlating the lists from the XE325 and XE324 plates, we obtained lists of "mismatches" between the two plates. Many of the objects included will be artifacts of various sorts. Some detections on the XE324 plate are not on the XE325 in the overlapping region, and vice versa. However, it seems that the XE325 plate has more transients.

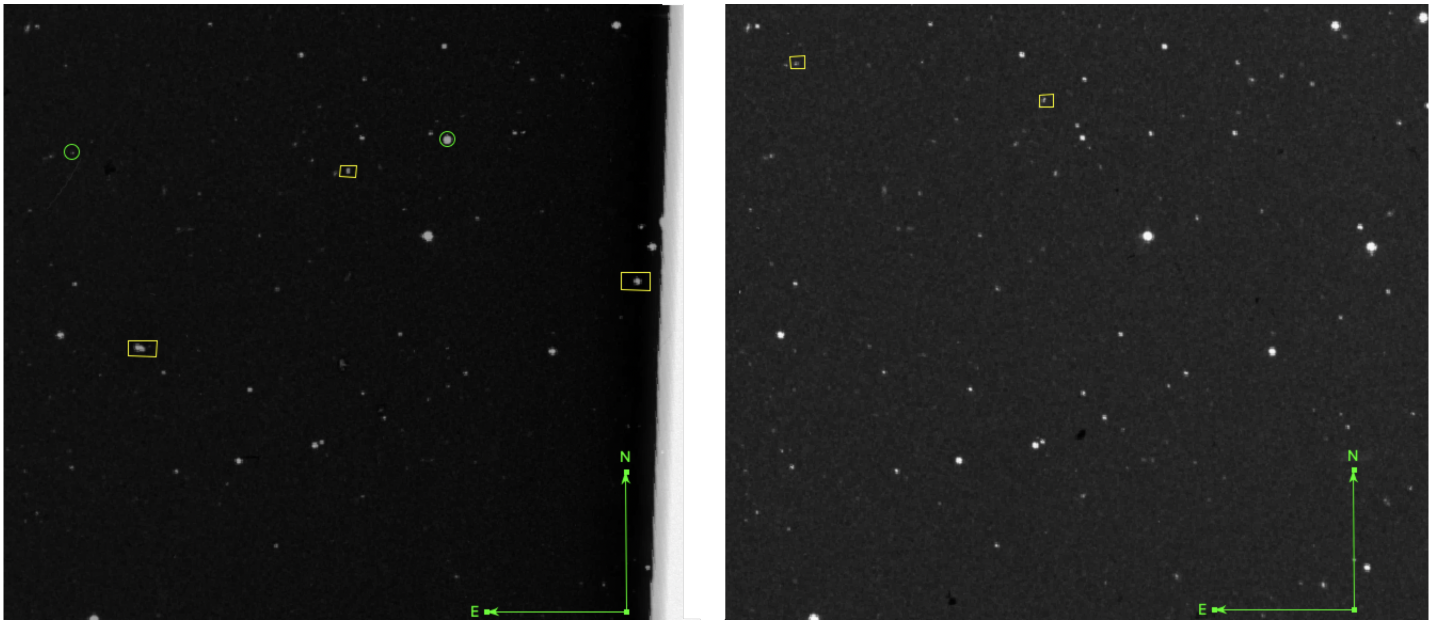

Figure 3. A bright transient inside a $0.25' * 0.25'$ **box.** The transient is located at (Ra, Dec) = 212.5714, 27.0909. The left image shows the field on the 12th of April 1950. The right image shows the field on the 18th of April 1950. It is also not seen 30 minutes earlier in the blue image (XO 325). The bright star is never seen again. Possible transients are marked with green circles, and artifacts with yellow squares. The bright transient is very close to the edge of the photographic plate (see the right cut-off edge).

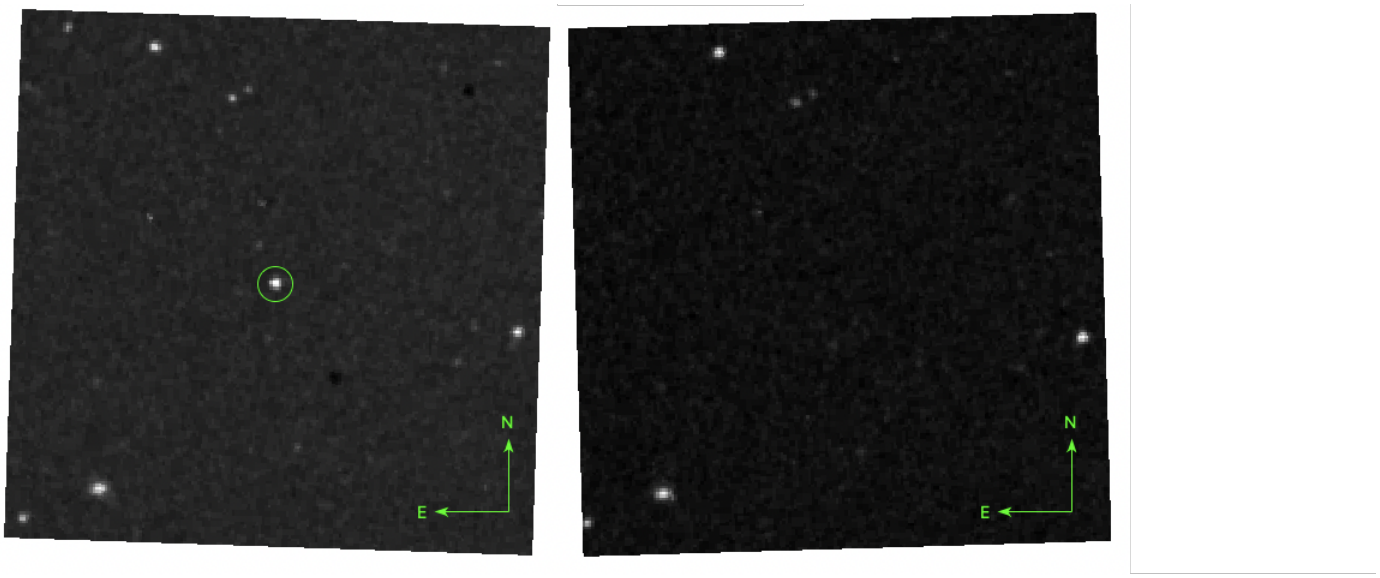

Figure 4. A bright transient inside a $5' * 5'$ **box.** The transient is located at (Ra, Dec) = 212.9083, 28.8368. The left image shows the field on the 12th of April 1950. The right image shows the field on the 18th of April 1950. It is also not seen 30 minutes earlier in the blue image (XO 325).

We checked how many bright objects in this region at XE325 have r Gaia magnitudes $r < 17$ but have no counterpart on XE324 (down to $r < 17$). This yielded 33 objects that we visually inspect. Inspection showed that 20 are obvious artifacts. Four are normal stars the photometry code failed to identify. About 10% of objects were missed due to difficulties in automatically detecting objects in crowded areas. We arrived at a short list of 9 potential transients, that need to be carefully inspected, listed in Table 2.

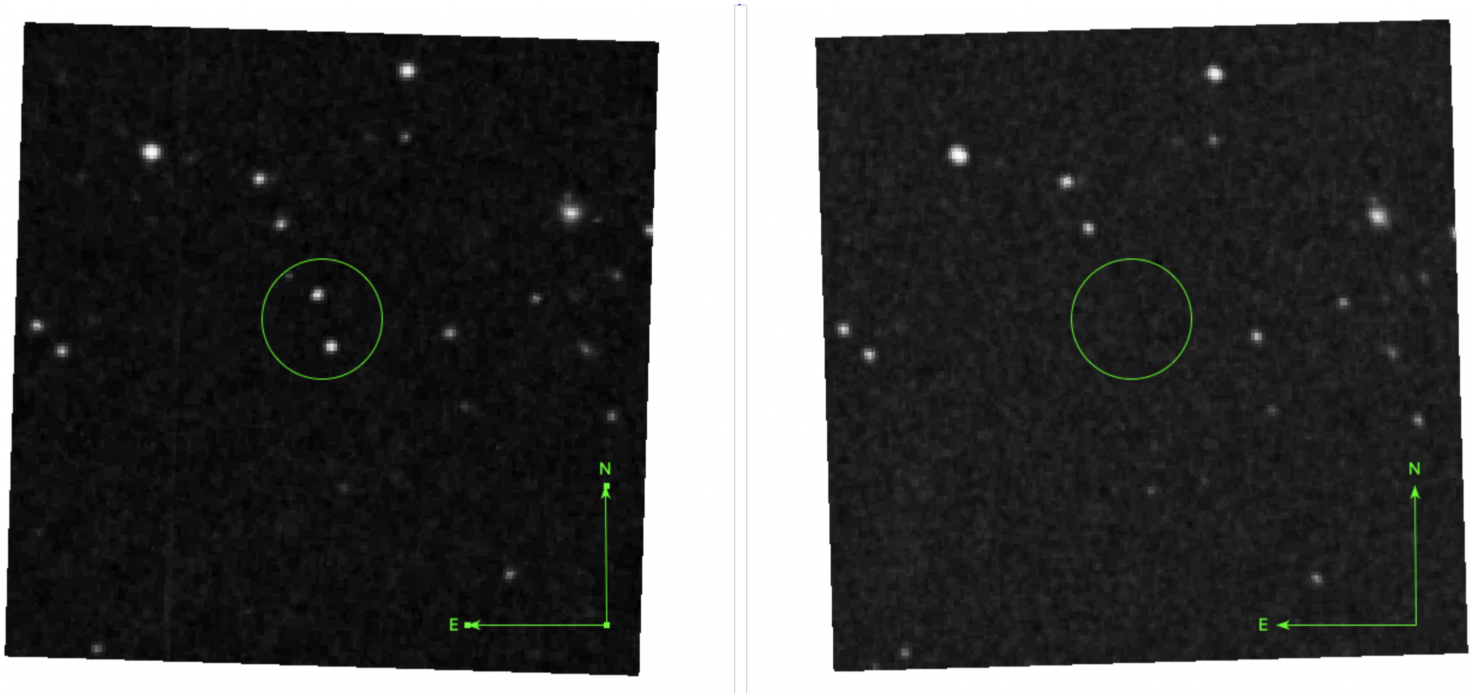

Figure 5. Two bright transients inside a $5' * 5'$ box. The transient is located at (Ra, Dec) = 212.82222, 32.075298. The left image shows the field on the 12th of April 1950. The right image shows the field on the 18th of April 1950. It is also not seen 30 minutes earlier in the blue image (XO 325).

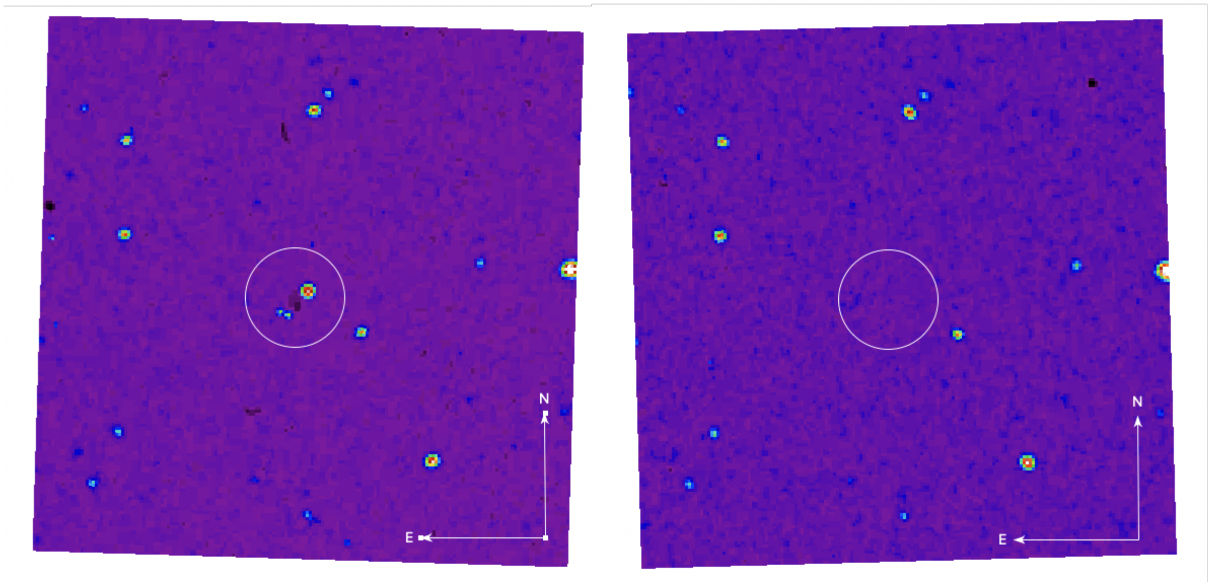

Figure 6. A bright transient inside a $5' * 5'$ box. The transient is located at (ra,dec) = 212.83594, 26.944296. The left image shows the field on the 12th of April 1950. The right image shows the field on the 18th of April 1950. The transient is located near a dark patch, which is easier to see with the *sls* colour map in DS9. It is also not seen 30 minutes earlier in the blue image (XO 325).

We used DS9 to inspect the transients in Table 2. Something with the appearance of a triple transient is located on top of a dark patch of the plate, which clearly indicates it is an artifact. Another two candidates are located near dark patches. Removing these, about 4 possible bright transients with $r < 17$ remain. That means that 29 out of 33 detections were artifacts.

Table 1. Plates from STSci. They are ordered in time of exposure, as reported from STSci.

Samples			
Plate	Date	Ra	Dec
XO445	1950-04-11	241.4761	17.6448
XE445	1950-04-11	241.4761	17.6448
X0325	1950-04-12	209.0709	29.3602
XE325	1950-04-12	209.0709	29.3602
XE384	1950-04-12	228.9373	23.5156
XO384	1950-04-12	228.9373	23.5156
XE324	1950-04-18	228.9373	23.5156

A particularly interesting case is a double system located near (ra, dec)= 212.9083, 28.8368. We show it in Figure 4. Here, two stars that look very real and similar to real, confirmed stars in their vicinity, simultaneously appear and vanish. We use this case as an example of anomalies that the VASCO citizen science project (6) searches for .

Table 2. Nine additional candidates from the photometry run. The detections that look real, are marked with an asterisk (*). Four detections appear real.

Samples			
ra	dec	r	comment
212.79430	26.340967	13.8	triple system on top of dark patch.
212.80032	26.339046	16.21	”
212.80287	26.343384	14.34	”
212.81104	30.015404	16.38	Close to dark patch.
212.82222	32.075298	16.57	Looks real (*).
212.83594	26.944296	15.52	Looks real, but nearby dark patch (*).
212.86185	27.412319	16.96	Slight elongation?
212.90825	28.836828	16.984	Two stars vanish (*).
213.00134	27.449156	15.847	Two stars vanish (*).

A.7. *Additional tests*

A.7.1. *Different spectral sensitivity of the filters?*

If the POSS-1 red emulsion has larger sensitivity to higher wavelengths than the POSS-2 red images, one may expect to possibly find more objects in the POSS-1 red images over the POSS-2 red images. That could reveal a number of brown dwarfs in the POSS-1 red images that are lost from POSS-2 red images. Our coordinate, however, has two images of exactly the same kind (same spectral sensitivity) and exposure time recorded in the DSS in 1950. If we compare the XE324 (18th of April 1950) and XE325 plate (12th of April 1950), we find that the XE324 plate lacks the 9 sources entirely. We also double checked by comparing with the Pan-STARRS y-band image that peaks around 1 μm, and also does not reveal any additional 9 objects.

A.7.2. *Scanning effects*

Another possibility is that the scanning procedures of the images in DSS created the false stars. The scanning procedure includes a glass cover put on top of the photographic plates. The glass cover has scratches and dirt that can appear like “transients”, see Fig 7. By using an independent scanning of the photographic plates, we can check whether the objects are still there or not, or if they have any deformities.

We use the SuperCosmos catalogue that has higher resolution scans and compare with our DSS **digitization** of the same field. Any object in the POSS-I r image that arise from scanning is marked in purple throughout the paper. Here, we can see that the nine simultaneous transients are clearly visible in the high-resolution scans of the POSS-1 red images also in the SuperCosmos catalogue. Thus, the scanning procedure of DSS is not causing our nine simultaneous transients.

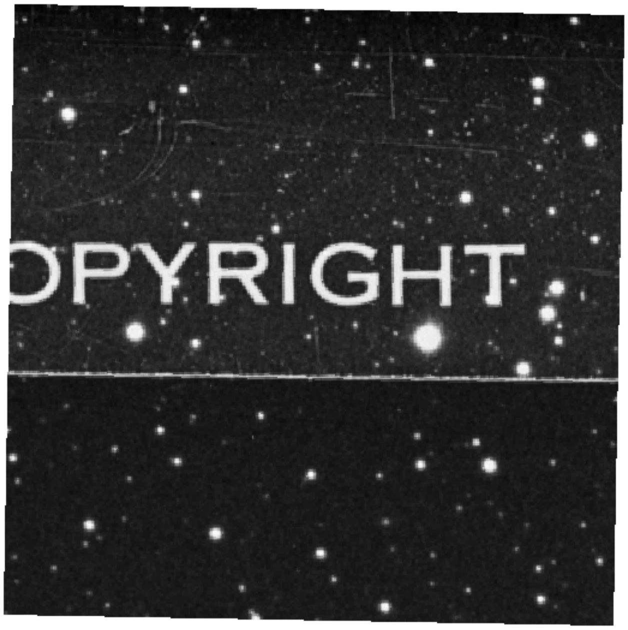

Figure 7. Scanning defects in the DSS. The scanning process of the plates includes a glass cover on top of the plate. We show the effect of the glass on top of a photographic plate. As can be seen, scratches and dirt can create false "transients".

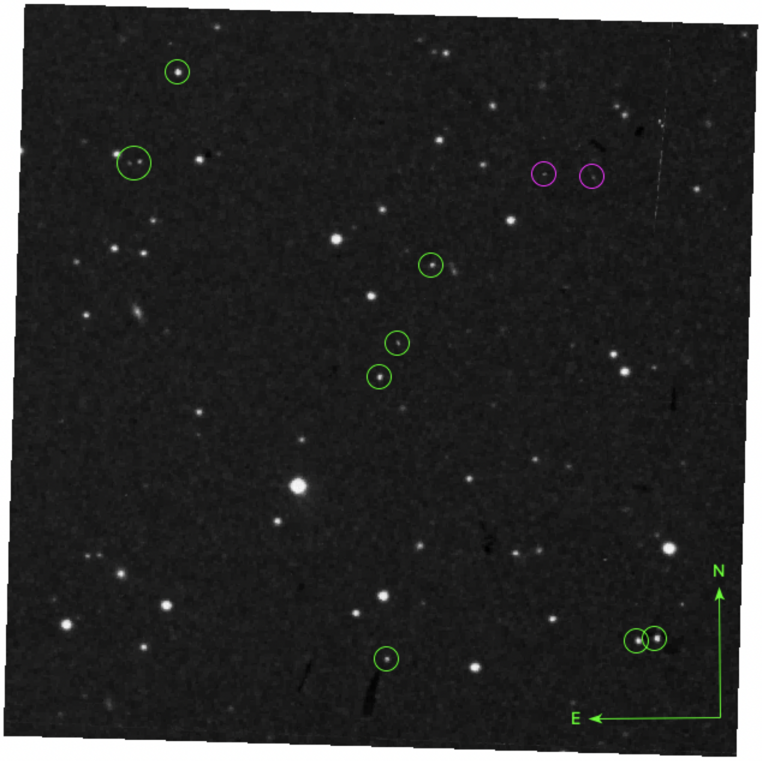

Figure 8. A higher resolution view. We show a higher resolution view of the same field in Supercosmos. Purple circles are artifacts during the scanning process. Note the clarity of transients 1, 7 and 8.

A.7.3. *Many artifacts lead to vanished stars?*

One may consider the hypothesis that the more artifacts, the more transients. Maybe, the transients are pure plate defects, even. However, the field near the bright beautiful transient shown in Figure 3, is rather clean and has few visible artifacts nearby (except the edge of the plate). We see these tendencies at more places, which speaks against a correlation between transients and artifacts.

A.7.4. *Double-exposed images?*

No human endeavour is free from errors and mistakes. Let us make the following thought experiment. A young observer is given the task to scan the sky one particular night during the First Palomar Survey. By mistake, the observer incidentally points the telescope wrongly and exposes the photographic plates for just a few seconds to the wrong field, exclaiming a “Sigh!” and as soon as possible our young observer corrects the error and the position, but without unfortunately changing to new plates. After the few seconds of exposure, the most prominent stars from the “wrong” field leave their traces on the corrected image. How could we find out if such a scenario explains our 9 simultaneous transients? The easiest way would, of course, be to knock on the door of the observer about 70 years later, and ask openly and loudly: “Has such an incident happened on your watch?” But given the current Covid-19 pandemics, the strategy has evidently failed.

Thus, what remains for us to do is performing a few tests to check whether the double exposure scenario is plausible or not. If yes, the additional transients may be spread evenly over the whole plate. Otherwise, we may expect them to be concentrated just in one area, assuming a totally open shutter. In the experiment, we shall only consider the DSS digitalisations of the plates, where we do not separate between scanning artifacts and transients. This is because not every region of the XE325 plate has the equivalent SuperCosmos scan.

We see that we can find more “vanishing objects” up to a box of maybe 20×20 or 25×25 arcmin. What happens if we look count the number of “vanishing objects” at different distances from the central point? We use the DSS Plate Finder to examine the fields. We examine four fields, $10' \times 10'$, displaced roughly 7’ N, S, E, and W from the center field (212.9291361,26.8310848). There is a little overlap with the central image. We also examined one additional image located $14'$ north of the center field. The results are summed in Table 3. It appears, like there is a larger concentration of transients in the “center field” where many transients have disappeared at the same time. This speaks against a double exposure with a completely open shutter.

Another way of testing the idea, is to see if any of the bright stars from the field observed right before or after, can be seen as a “bright transient” in XE325. We first noted, that the blue plate was taken right before the red plate of exactly the same field. However, the blue plate was the first image of the night. The previous night XE445 had been the final observation, and after XE325, the field of XE384 was observed. The bright stars in XE445 and XE384 appear to have left no trace in the plate of XE325, which again speaks against a double exposure (at least with a completely open shutter) of at least these two fields.

While these two tests cannot disprove the idea of a double exposure, we do not find any arguments in favour, either.

Table 3. The number of transients in nearby fields. Here, we have not separated scanning artifacts from other transients.

Samples	
Field	Number of Vanished Stars
Center field	13
7 arcmin north	4
14 arcmin north	5
7 arcmin south	2
1 min east	1
1 min west	0

A.7.5. *Cosmic rays?*

Non-astronomical effects apart from instrumental errors may arise from elementary particles. Alpha, beta or gamma particles can certainly leave traces in a photographic plate (7; 8; 9), but it is doubtful that the Kodak 103-aE red emulsions were sensitive enough to detect them, and even more, leave star-like brightness profiles of varying intensities.

The quantum efficiency of regular photographic plates is 1-2%, as compared to 80-90% of the CCDs. The many well-known reasons on how to produce an optical flash on a photographic plate used in astronomy (10) do not include cosmic rays. We can exclude muons and natural radioactivity, as these particles hit the photographic plates at a steady rate. The given 10×10 arcmin^2 region was certainly hit by thousands of elementary particles during the 50 minutes of exposure, and experienced several orders of magnitude more of hits during the months of storage, which disagrees with a few plates having extra stars (had muons and natural radioactivity caused extra stars, the photographic plates would be full of them). Moreover, the elementary particles from afar must hit the plate from all inclination angles, and leave even more elongated shapes and tracks inside the same region, although the shapes might be debatable (see Supp Info, Section A.7.6).

But how about a rare cosmic ray shower produced a few centimeters above the plate? Such a shower of particles carrying a big range of energies, conserving its momentum, would would spray within a cone toward the plate, intersecting the emulsion within a roughly circular region. One of the main problems with this hypothesis, is that while the photographic plate only registers the rare cosmic ray shower event, it must also be blind to all the other millions of cosmic ray particles of all possible energy ranges that must have hit the same region during months or years storage during or before exposure (unless the photographic plates were stored in lead boxes). Another problem is that while the blue emulsions are more sensitive to high-energy particles than the red ones, the seven cases of multiple transients have all been found on the red plates. While not an impossible scenario, it seems excessively unlikely that the red simultaneous transients were caused by cosmic rays. It is therefore not surprizing, that not a single mention of cosmic ray-events can be found in the many searches for optical counterparts to GRBs on photographic plates in the literature, see e.g. (11; 12). In the latter work, 482 hours of exposure time resulted in not a single unexaplainable flash – water drops and dust grains were easily identified with a magnifying glass. The rest were satellite glints.

A.7.6. *Shape of a hit*

Could an elementary particle create PSF-like shapes? The Kodak emulsion has a thickness of only ~ 20 microns. As an example, if the particle enters the emulsion 30 degrees to the normal, the projected length of the "track" on the plate is $s = 20 * tan(30) = 10\mu$m. We also must assume that the particle deposits energy and exposes silver halide crystals along its track with a width of roughly 29 μm, comparable to the PSF width created by the atmospheric seeing, to mimic the width of a star image. The resulting exposed silver halide grains will resemble a star image but convolved with a 10 μm long line, thereby elongating the image to an oval shape with the long axis having length (adding in quadrature), $\sqrt{10^2 + 29^2} = 30.7\mu$m. Compared to the FWHM of the seeing PSF, the image will appear point-like.

This is however a pure geometric estimate, and were it complete, all PSF widths would always be the same. As in any scattering problem, other parameters such as energy also enters. It is beyond the scope of the paper to do a modeling of how the silver-halide crystals on the plate react to elementary particles.

A.7.7. *What are the other multiple transients?*

Among the ~ 100 VASCO transients, there are more cases of multiple transients (about 7 in total). Examining them, we see that some cases appear in what seems to be contaminated extended regions with very faint star-like objects. This includes the central objects at R=168.683437 and DEC=8.2918095, or another at RA=173.1065531 and DEC=5.3817889. In these regions, we see more objects with all kind of shapes appear inside a given region (figure "8", elongated spots, foggy spots, etc). For weak sources near the noise limit, it is significantly more difficult to separate real point sources from artifacts and plate defects, including scanning defects (see Section A.7.2). Maybe some of the faintest transients are indeed examples of exposure of the silver halide crystals due to contamination or degradation. At the same time, we see one case of multiple transients in a clean region of a plate, where three transients cluster in the very center (RA=131.0121324, DEC=57.9448585). Several of the of the "simultaneous transients" on the XE325 plate are quite luminous and star-like, see e.g. the transients in Figure 5 (Supp. Info.). For future searches of multiple transients, it may be wise to restrict the searches to stars with $r < 18$ as these ones have better eventual possibilities for detailed examination with a microscope.

A.8. *Astrophysical considerations*

Natural transient events are expected to be occur now and then within our images (13). The Large Synoptic Survey Telescope expects to find 100 000 short-lived (visible < day) per day, which corresponds to about 2.5 events per square degree per day.

Flares as those from M dwarfs, would produce blue much more light. A gas at 10 000 K will produce Balmer lights both in blue and red, and may be expected to be see on the blue plates as well, and will last longer than a few minutes. Flares will also have their significantly fainter dwarf counterpart observable in the infrared.

Possibly, stars with very strong and variable Hα emission and with variable extinction due to dense molecular clouds could produce some variability easier observable in the red band. Examples of such are Be stars (14).

The rate of microlensing from the OGLE-IV survey, that covers around 3000 square degrees around the Galactic center, is about 2000 events per year, which corresponds to about 0.67 microlensing events per square degree per year.

In a pair of plates, where each plate covers about $4*4$ square degrees as the POSS-1 XE325 plates, the expected upper limit of detectable microlensing events when comparing two plates is $0.67*4*4*\tau \sim 1.8$ events (assuming each microlensing events lasts 2 months so that $\tau \sim 1/6$). This is far lower than the number of transients in the XE325 plate.

We therefore also consider the rates of GRBs. The rate of finding GRBs is about 1 per day over the whole Sky, which excludes this hypothesis. The rate of finding a FRB is 1 per day per square degree, which means that each day 16 FRBs might be found in the XE325 plate. However, these events last at maximum a few hours. Perhaps, catching the afterglows in different phases, still makes it possible to record the many we've found. However, the transients in the XE325 plate cover a much smaller region of $10*10$ arcmin^2 (only a fraction of the entire plate), and also here the detectable number densities of our simultaneous transients are just too high.

To summarize, the rate of natural transients (2.5 events per square degree per day) makes the probability to discover 9 transients within a 30 minutes and $10*10$ arcmin^2, close to zero.

If the observed phenomenon is a real and natural astrophysical phenomenon, one must also consider how the simultaneous transients can be so well timed when extended over such a large region of the sky. We certainly, see it over a $10*10$ arcmin^2 and the phenomenon may extended even larger up to one or two degrees in each direction.

In order for the "dots" to have a communication in turning on or off, they must be physically separation d less than 30 light minutes, so that $d_s < 5.4*10^{11}$ m. The distance r to this phenomenon, can then be calculates as

$$r = d_s/\omega \tag{A1}$$

where ω is expressed in radians. For $\omega = 10'$, this corresponds to $r = 1.9*10^{14}$ meters or 0.02 light years which is inside our Solar System. If the transient phenomenon extends up to two degrees, the distance to the phenomenon is less than a light day away.

A.8.1. *Calculating the multiple transient rate*

We can easily estimate the number of "multiple transient", expected in the POSS-I data. We use the 7 cases we have. Given that the detections happened after that 15% of all 150 000 candidates were visually examined in (15), the expected number of multiple transients in POSS-I is at least ~ 45 and represents everything the survey encountered during its 16 years of surveillance between 1949 - 1966, which corresponds to 936 "red" fields, each exposed for ~ 50 minutes each. That means about ~ 780 total hours of exposure covering roughly 80% of the sky, each exposure of $6*6$ sq. degrees. About 45 cases during 780 hours of exposure and correcting for the sky coverage with a factor of 1/0.80, means that the discovery rate of pre-satellite glints is $\sim 0.058/0.80$ per hour. That is about ~ 0.07 hour^{-1} sky^{-1} i.e. negligible in comparison to the current number of satellite glints an astronomer at a telescope near the equator sees today, ~ 1800 hour^{-1} sky^{-1} (16; 17). Of these, we expect that less than half will show at least three transients following a straight line.

REFERENCES

[1] Cepa J., Aguiar M., Escalera V. G., Gonzalez-Serrano I., Joven-Alvarez E., Peraza L., Rasilla J. L., et al., 2000, SPIE, 4008, 623. doi:10.1117/12.395520

[2] Cepa J., 2010, ASSP, 14, 15. doi:10.1007/978-3-642-11250-8_2

[3] Bertin et al. 2002, ASP Conference Series, Vol. 281, D.A. Bohlender, D. Durand, and T.H. Handley, eds., 228

[4] Bertin E., Arnouts S., 1996, A&AS, 117, 393

[5] Andruk V., Villarroel B., Streblyanska, et al. (to be submitted)

[6] Villarroel B., Pelckmans, K. Solano E. et al., arXiv: 2009.10813, arXiv: 2009.10813

[7] Becquerel H., "Sur les radiations émises par phosphorescence", 1986, Comptes Rendus, 122, 420

[8]Kinoshita S., 1910, Proceedings of the Royal Society of London, 83, 432

[9]Sisefsky J., 1961, Science, 133, 735

[10]Schaefer B.E., 1988, ApJ, 337, 927

[11]Schaefer B.E., 1981, Nature, 294, 722

[12]Schaefer B.E., Barber M., Brooks J.J. et al., 1987, ApJ, 320, 398

[13]Bellm E.C., 2016, The Astronomical Society of the Pacific, 966, 128

[14]Hernández J., Calvet N., Briceno C., Hartmann & Berlind P., 2003, Astronomical Journal, 127, 1682

[15]Villarroel B., Soodla J., Comerón S. et al, 2020, Astronomical Journal, 159, 8

[16]McDowell J.C., 2020, ApjL, 892, L36

[17]Corbett et al. 2020, arXiv: 2011.02495
